# Regulation of bone mass through pineal‐derived melatonin‐MT2 receptor pathway

**DOI:** 10.1111/jpi.12423

**Published:** 2017-06-20

**Authors:** Kunal Sharan, Kirsty Lewis, Takahisa Furukawa, Vijay K. Yadav

**Affiliations:** ^1^ Systems Biology of Bone Department of Mouse and Zebrafish Genetics Wellcome Trust Sanger Institute Cambridge UK; ^2^ Institute of Protein Research Osaka University Osaka Japan; ^3^ Metabolic Research Laboratory National Institute of Immunology New Delhi India; ^4^ Present address: Department of Molecular Nutrition CSIR‐Central Food Technological Research Institute Mysore India

**Keywords:** bone, melatonin, osteoblasts, osteoporosis, tryptophan

## Abstract

Tryptophan, an essential amino acid through a series of enzymatic reactions gives rise to various metabolites, viz. serotonin and melatonin, that regulate distinct biological functions. We show here that tryptophan metabolism in the pineal gland favors bone mass accrual through production of melatonin, a pineal‐derived neurohormone. Pineal gland‐specific deletion of *Tph1*, the enzyme that catalyzes the first step in the melatonin biosynthesis lead to a decrease in melatonin levels and a low bone mass due to an isolated decrease in bone formation while bone resorption parameters remained unaffected. Skeletal analysis of the mice deficient in *MT1* or *MT2* melatonin receptors showed a low bone mass in *MT2−/−* mice while *MT1−/−* mice had a normal bone mass compared to the WT mice. This low bone mass in the *MT2−/−* mice was due to an isolated decrease in osteoblast numbers and bone formation. In vitro assays of the osteoblast cultures derived from the *MT1−/−* and *MT2−/−* mice showed a cell intrinsic defect in the proliferation, differentiation and mineralization abilities of *MT2−/−* osteoblasts compared to WT counterparts, and the mutant cells did not respond to melatonin addition. Finally, we demonstrate that daily oral administration of melatonin can increase bone accrual during growth and can cure ovariectomy‐induced structural and functional degeneration of bone by specifically increasing bone formation. By identifying pineal‐derived melatonin as a regulator of bone mass through MT2 receptors, this study expands the role played by tryptophan derivatives in the regulation of bone mass and underscores its therapeutic relevance in postmenopausal osteoporosis.

## 1 INTRODUCTION

Skeleton is one of the largest organ systems in the vertebrate body and performs multiple mechanical, hematopoietic and endocrine functions.^1^ Adult skeleton is continuously renewed through bone remodeling, a homeostatic process regulated by a fine balance in the actions of two cell types: osteoblasts that make new bone and osteoclasts that resorb old bone.^2^, ^3^, ^4^, ^5^ Bone remodeling is regulated by paracrine, endocrine and neuroendocrine signals, and a dysregulation in any of these pathways affects skeletal physiology.^6^, ^7^ Despite significant advances in our understanding of skeletal (patho‐)physiology in the last 20 years, only one anabolic therapy has reached the clinic that can effectively increase bone mass for at least up to 2 years in humans, ie, intermittent injections of parathyroid hormone (iPTH).^8^ The challenge therefore remains to identify additional anabolic drug targets that can complement and/or are better than iPTH to prevent or cure osteoporosis in humans.

Melatonin is a bioamine generated in the pineal gland, but is also produced in several peripheral sites especially the gut.^9^, ^10^, ^11^, ^12^ Melatonin is synthesized from the essential amino acid tryptophan following two pathways—the classical or an alternate pathway.^9^, ^10^, ^12^ The classical biosynthetic pathway is the predominant pathway through which melatonin is synthesized in the mammalian pineal gland, and it involves four consecutive steps.^9^, ^10^, ^12^ Tryptophan is first converted, through the action of tryptophan hydroxylase 1 (Tph1) into 5‐hydroxytryptophan (Step 1), which is then converted into serotonin (Step 2) by the action of aromatic amino acid decarboxylase (AADC). Serotonin is next converted through the action of arylalkylamine *N*‐acetyltransferase (AANAT) into *N*‐acetylserotonin (Step 3), which then serves as a substrate for the enzyme *N*‐acetylserotonin *O*‐methyltransferase (ASMT) that converts it to melatonin.^9^, ^10^, ^12^, ^13^ An alternate pathway has been proposed recently that may play an important role in the production of melatonin in the peripheral tissues in which serotonin is first *O*‐methylated and the resulting 5‐methoxytryptamine is then *N*‐acetylated to produce melatonin.^12^ Melatonin produced by the pineal gland is a neurohormone that is released into the circulation and regulates a variety of physiological processes by either acting through cell surface receptors, melatonin receptor 1A (MT1)/melatonin receptor 1B (MT2), or by acting directly as an antioxidant in the cells.^14^, ^15^, ^16^, ^17^

Melatonin is known to regulate many functions including circadian rhythm, reproduction, temperature, immune system and the cardiovasculature, and therapeutic avenues are being explored for melatonin in the treatment of many diseases.^18^, ^19^, ^20^, ^21^, ^22^, ^23^ Melatonin has been shown to influence osteoblast functions in vitro and enhance bone regeneration in calvarial defect in explant assays.^24^, ^25^ However, the cellular origin of melatonin synthesis and its potential as a regulator of bone mass accrual in the in vivo context have not been thoroughly investigated.^7^, ^26^ Given the biology surrounding melatonin and its receptors, and its clinical relevance as underscored above, it is of utmost importance to understand the cellular origin and the mechanisms through which melatonin affects bone mass.

Here we show that pineal‐derived melatonin (PDM) positively regulates bone mass. Cell‐specific gene deletion and oral feeding of melatonin in loss of function models show that melatonin regulation of bone mass accrual occurs through MT2 receptor. MT2 receptor expressed on the osteoblasts regulates, in a cell autonomous fashion, *Cyclin* expression to increase osteoblasts proliferation and function. We further show that oral melatonin treatment can cure gonadectomy‐induced architectural deterioration and biomechanical properties of bones in rodents. These results reveal an unanticipated molecular link between pineal and bone through alterations in serum melatonin levels and identify novel therapeutic avenues for treating postmenopausal osteoporosis.

## 2 METHODS

### 2.1 Animals

We performed all procedures on mice conforming to the ethical regulations of National Institute of Immunology, Wellcome Trust Sanger Institute and the UK Home Office. To generate pineal‐specific deletion (*pineal−/−*), *Tph1* ^*flox*/+^ mice were crossed with *Crx‐Cre mice* to generate *pineal+/−* mice, and their progeny were intercrossed to obtain *Tph1*_*pineal*_ *−/−* mice. Generation of *Tph1* ^*fl*/*fl*^, *MT1−/−*,* MT2−/−* and *Crx‐Cre* mice was previously reported.^27^, ^28^, ^29^, ^30^ We obtained C3H/HeJ and C57Bl6 mice from the inbred wild‐type colonies maintained at the Wellcome Trust Sanger Institute and National Institute of Immunology research support facility.

### 2.2 Curative regimen in mice

We subjected 6‐weeks‐old virgin C57Bl/6J female mice to either bilateral ovariectomy or sham operation. We next treated mice for 6 weeks starting 6 weeks after ovariectomy with melatonin (10 or 100 mg/kg body weight/d) or vehicle by oral gavage. At the end of experiments, animals were culled and the skeleton was fixed in 4% neutral buffered formalin or frozen for mechanical testing. Right tibia and lumbar vertebral column were collected in 70% alcohol and were processed for μCT or histomorphometric analysis.

### 2.3 Histology and μCT of bones

Histological analyses were performed on vertebral column specimens collected from 3‐month‐old mice (unless otherwise stated) using undecalcified sections stained with von Kossa/von Gieson or other reagents.^30^, ^31^ Static and dynamic histomorphometric analyses were performed according to standard protocols using the Osteomeasure Analysis System (Osteometrics, Atlanta, GA, USA). μCT scanning of excised tibia was carried out as described before^32^ using a Skyscan 1172 μCT system with standard software provided by the manufacturer.

### 2.4 Cell cultures

#### 2.4.1 Bromodeoxy uridine incorporation‐based osteoblast proliferation assay

Primary calvarial osteoblasts from WT or mutant mice (*MT1−/−* or *MT2−/−*) were used for cell proliferation assays. Briefly, at 70%‐80% confluence cells were trypsinized, and 5000 cells/well were seeded in 96‐well plate in α‐MEM supplemented with 10% fetal bovine serum (FBS). After 24 hours, cells were left for another 24 hours in α‐MEM supplemented with 0.5% FBS. Four hours before the end of 24 hours stimulation period, the cells were pulsed with bromodeoxy uridine (BrdU), and BrdU incorporation was measured using an ELISA kit (Roche, Indianapolis, IN, USA).

#### 2.4.2 Alkaline phosphatase activity assays

Primary calvarial osteoblasts from WT and or mutant mice (*MT1−/−* or *MT2−/−*) at 70%‐80% confluence were trypsinized and 5000 cells/well were seeded in 96‐well plates. Cells from WT and mutant (*MT1−/−* or *MT2−/−*) mice were cultured for 48 hours in an osteoblast differentiation medium containing α‐MEM supplemented with 10% FBS, 10 mmol/L β‐glycerophosphate, 50 mg/mL of ascorbic acid and 1% penicillin/streptomycin. At the end of incubation period, total alkaline phosphatase (ALP) activity was measured by an alkaline phosphatase assay kit using *p*‐nitrophenylphosphate (PNPP) as substrate, and color absorbance was read at 405 nm (SensoLyte® pNPP Alkaline Phosphatase Assay kit; AnaSpec, Inc., Fremont, CA, USA).

#### 2.4.3 Osteoblast mineralization assays

Primary osteoblasts from WT or mutant (*MT1−/−* or *MT2−/−*) mice were seeded in 12‐well plates (25 000 cells/well) in osteoblast differentiation medium and cultured for 21 days with a medium change every 48 hours. At the end of the experiment, cells were washed with PBS and fixed with 4% paraformaldehyde for 15 minutes. Alizarin red‐S was used for staining, visualizing mineralized nodules and quantification.

### 2.5 Bioassays

Serum melatonin levels were quantified by ELISA (melatonin kit; IBL International, Hamburg, Germany). In the case of genetic ablation of *Tph1* on C3H/HeJ or 129/SvJ, background blood was collected between 400 and 600 hours while in the case of pharmacological experiments with exogenous melatonin treatment blood was collected between 1400 and 1600 hours. Blood was allowed to clot on ice before centrifugation to collect the clear supernatant.

### 2.6 Molecular studies

Total RNA was isolated from either primary calvarial osteoblasts or long bones flushed of bone marrow. Real‐time PCR was performed on DNase I‐treated total RNA converted to cDNA using appropriate primers and standard protocols. β‐Actin amplification was used as an internal control. Quantitative RT‐PCR primer sequences used in our study were as follows. *MT1* (forward primer [F]: TGTGTACCGCAACAAGAAGC; reverse primer [R]: GAGGCTGTGGCAAATGTAGC), *MT2* (F: CCTCTCAGTGCTCAGGAACC; R: CAGAGCCAATGACACTCAGG), *Alp* (F: CAGAGCCAATGACACTCAGG; R: TGGCTACATTGGTGTTGAGC), *Runx2* (F: AGATGGGACTGTGGTTACCG; R: TGGTCAAGGTGAAACTCTTGC), *Osx* (F: CCTCTGCGGGACTCAACAAC; R: TGCCTGGACCTGGTGAGATG), *Trap* (F: AAACCCGTGAGGAAGAGAGC; R: GGACATTTTCCTCCTTCTTGG), *RankL* (F: GGAAGCGTACCTACAGACTA; R: AGTACGTCGCATCTTGATCC), *Opg* (F: ACAGTTTGCCTGGGACCAAA; R: TCACAGAGGTCAATGTCTTGGA), *Ccnd1* (F: GCGTACCCTGACACCAATCTC; R: CTCCTCTTCGCACTTCTGCTC), *Ccnd2* (F: GAGTGGGAACTGGTAGTGTTG; R: CGCACAGAGCGATGAAGGT), *Ccne1* (F: GTGGCTCCGACCTTTCAGTC; R: CACAGTCTTGTCAATCTTGGCA), *Ccne2* (F: ATGTCAAGACGCAGCCGTTTA; R: GCTGATTCCTCCAGACAGTACA), *Ccnf* (F: GTAGGCGATAGGTCATACGGA; R: ACAATGGATCACTACCCCGTG) and *β‐Actin* (F: GACCTCTATGCCAACACAGT; R: AGTACTTGCGCTCAGGAGGA). *OPG* and *Rankl* expression was first normalized with *β‐Actin* levels, and then, ratio *OPG*/*Rankl* was expressed as relative to the WT sample value. Genotypes of all the mice were determined by genomic DNA PCR. Genotyping primers used in our studies to genotype different mutant mice were as follows. *MT1−/−* mice (1A1: GAGTCCAAGTTGCTGGGCAG; 1A2: GAAGTTTTCTCAGTGTCCCGC; 1A3: CCAGCTCATTCCTCCACTCAT), *MT2−/−* mice (2B1: CCGCAGCCTCTTCTTAACAC; 2B2: CTGCCACTGAGGACAGAACA; 2B3: GCCAGAGGCCACTTGTGTAG), *Crx‐Cre* (Cre1: GACGATGCAACGAGTGATGA; Cre2: AGCATTGCTGTCACTTGGTC) and *Tph1*Floxed mice (F: GGATCCTAACCGAGTGTTCC; R: GCACACCACCAACTCTTTCC).

### 2.7 Statistical analyses

**Figure 1:**
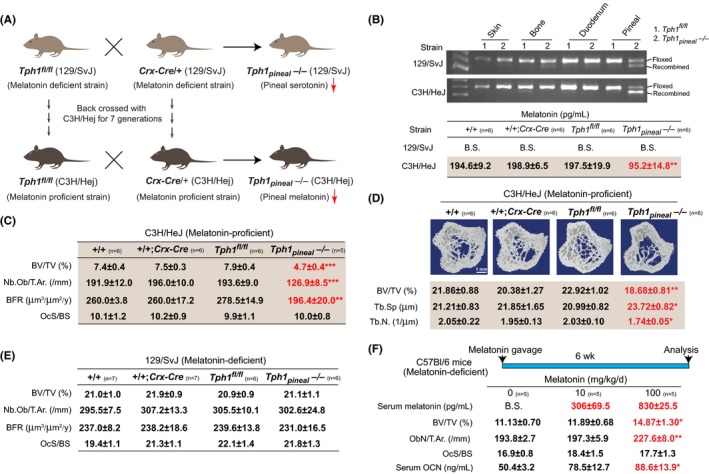
Pineal‐derived melatonin positively regulates bone mass. (A) Schematic representation of the breeding strategy to obtain *Tph1*_*pineal*_ *−/−* on 129/SvJ (melatonin‐deficient) and C3H/HeJ (melatonin‐proficient) genetic backgrounds. (B) Recombination PCR for *Tph1* in tissues and serum melatonin levels in different genotypes. (C) Histological analysis of vertebra. (D) μCT analysis of tibia in 12‐wk‐old +/+, +/+; *Crx‐Cre, Tph1* ^*fl/fl*^ and *Tph1*_*pineal*_ *−/−* mice on C3H/HeJ background. (E) Histological analysis of vertebra of 12‐wk‐old WT, +/+; *Crx‐Cre, Tph1* ^*fl/fl*^ and *Tph1*_*pineal*_ *−/−* mice on 129/SvJ background. (F) Serum melatonin, vertebral histomorphometry and serum osteocalcin levels in WT C57BL/6 mice treated with 0, 10 or 100 mg/kg/d melatonin for 6 wk. n for each group is indicated within each panel. Values are mean±SEM. Following symbols have been used to indicate level of significance: *P<.05; **P<.01; ***P<.001 compared to WT or control samples. ns, not significant. See also Fig. S1

**Figure 2:**
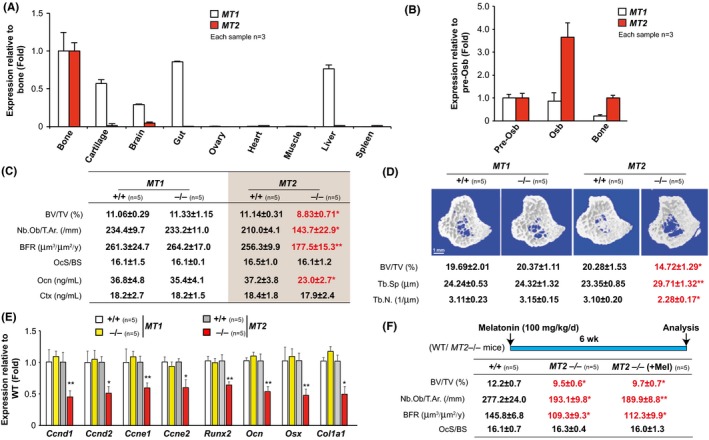
Melatonin promotes bone mass through MT2. (A) Real‐time PCR analysis of *MT1* and *MT2* expression in different tissues of the WT mice. (B) Real‐time PCR analysis of *MT1* and *MT2* in calvarial preosteoblast, differentiated osteoblast and long bone of the WT mice. (C) Vertebral histomorphometry and serum levels of osteocalcin (Ocn) and Ctx in *MT1+/+*,*MT1−/−, MT2+/+* and *MT2−/−* mice. (D) μCT analysis of tibia in *MT1+/+*,*MT1−/−, MT2+/+* and *MT2−/−* mice. (E) Real‐time PCR analysis of *Cyclins* and osteoblast marker genes in the tibia of *MT1+/+*,*MT1−/−, MT2+/+* and *MT2−/−* mice. (F) Vertebral histomorphometry of vehicle or melatonin‐treated +/+ and *MT2−/−* mice. n for each group is indicated within each panel. Values are mean±SEM. Following symbols have been used to indicate level of significance: *P<.05; **P<.01; ***P<.001 compared to WT or control samples. ns, not significant. See also Fig. S2

**Figure 3:**
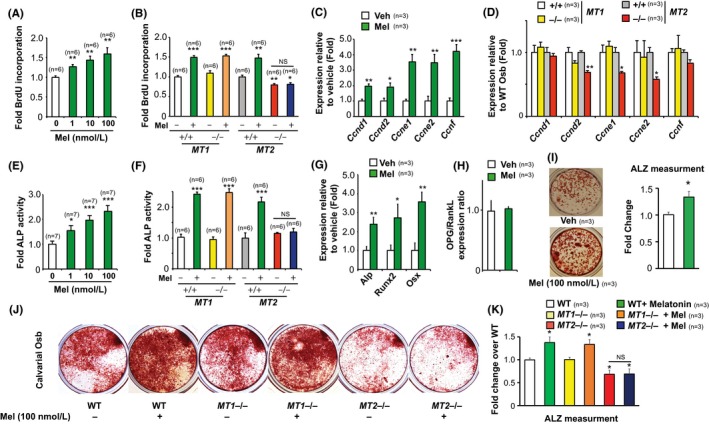
Melatonin directly regulates osteoblast functions through MT2 receptor. (A) BrdU incorporation assay in melatonin‐treated WT calvarial osteoblasts. (B) BrdU incorporation assay in *MT1+/+*,*MT1−/−, MT2+/+* and *MT2−/−* osteoblasts treated with vehicle or melatonin. (C, D) Real‐time PCR analysis of *Cyclins* in vehicle or melatonin‐treated WT osteoblasts (C) and in *MT1+/+*,*MT1−/−, MT2+/+* and *MT2−/−* osteoblast cells (D). (E) Alkaline phosphatase activity in WT osteoblasts treated with melatonin at different concentrations. (F) Alkaline phosphatase activity in *MT1+/+*,*MT1−/−, MT2+/+* and *MT2−/−* osteoblasts treated with vehicle or melatonin. (G) Real‐time PCR analysis of osteoblast marker genes and (H) OPG/RankL expression ratio in melatonin‐treated WT osteoblasts. (I) Alizarin red staining and quantification in WT cells treated with melatonin for 21 d. (J, K) Alizarin red staining (J) and quantification (K) in the calvarial osteoblast cells from *MT1+/+*,*MT1−/−, MT2+/+* and *MT2−/−* mice differentiated toward mineralizing osteoblasts. n for each group is indicated within each panel. Values are mean±SEM. Following symbols have been used to indicate level of significance: *P<.05; **P<.01; ***P<.001 compared to WT or control samples. ns, not significant. See also Fig. S3

**Figure 4:**
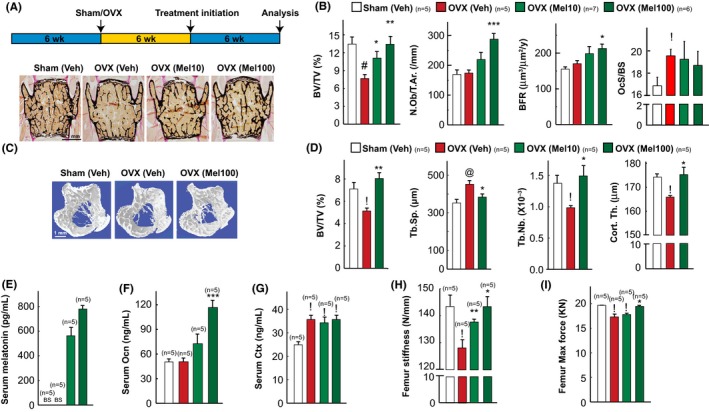
Daily melatonin treatment cures ovariectomy‐induced osteoporosis. (A) Schematic representation of the experimental protocol used and representative images of von Kossa stained vertebral sections of sham/OVX mice treated with vehicle or melatonin at 10 or 100 mg/kg/d dose. (B) Vertebral histomorphometric analysis in sham and OVX mice treated with vehicle or melatonin at 10 or 100 mg/kg/d dose. (C, D) 3D μCT images of proximal tibia (C) and its microarchitectural parameters (D) in sham/OVX mice treated with vehicle or melatonin. (E‐G) Serum melatonin (E), osteocalcin (F) and Ctx levels (G) in sham/OVX mice treated with vehicle or melatonin. (H, I) Femur stiffness (H) and femur maximum force analysis (I) by three‐point bending test in sham/OVX mice treated with vehicle or melatonin. Values are mean±SEM. n for each group is indicated within each panel. Results were considered significant at *P*<.05. In all the panels, following symbols have been used to indicate different levels of significance *P<.05, **P<.01 and ***P<.001 as compared to OVX (vehicle) and ^!^ *P*<.05, ^@^ *P*<.01, ^#^ *P*<.001 as compared to sham (Veh)

Results are given as means±standard error. Statistical analysis was performed by Student's *t* test between two groups and one‐way ANOVA followed by Newman‐keuls post hoc test for more than two groups. Fold changes were calculated in gene expression compared to the vehicle‐treated cells or WT samples. Results were considered significant at *P*<.05. In all the panels in Figures 1, 2, 3 and Figs S1‐S3, following symbols have been used to indicate different levels of significance: *P<.05, **P<.01 and ***P<.001 vs WT or control samples. In Figure 4, following symbols have been used to indicate different levels of significance: *P<.05, **P<.01 and ***P<.001 as compared to OVX (vehicle); and ^!^ *P*<.05, ^@^ *P*<.01, ^#^ *P*<.001 as compared to sham (vehicle).

## 3 RESULTS

### 3.1 Pineal‐derived melatonin regulates bone mass

To understand whether pineal‐derived melatonin regulates bone mass, we first generated a pineal‐specific conditional loss of function model of melatonin. Laboratory mouse strains differ significantly in their ability to produce melatonin. We therefore carried out our analysis on two different genetic backgrounds of mice: one that has normal levels of melatonin (C3H/HeJ, melatonin‐proficient) and the other that has <1% of this level (129/SvJ, melatonin‐deficient) in their serum. To achieve *Tph1* ablation on C3H/HeJ genetic background, we backcrossed *Tph1* floxed mice and *Crx‐Cre* mice, a pineal‐specific *Cre* recombinase, which were on pure 129/SvJ background for seven generations on a C3H/HeJ background (Figure 1A). This backcrossing normalized the levels of melatonin in their serum to the one observed in pure C3H/HeJ mice, a melatonin‐proficient strain (Fig. S1A). These melatonin‐proficient *Tph1* floxed and *Crx‐Cre* mice were then intercrossed to generate *Tph1*_*pineal*_ *−/−* mice. *Tph1*_*pineal*_ *−/−* mice had a 65% reduction in *Tph1* expression in pineal while *Tph1* expression in skin, bone and gut was not affected compared to controls (Fig. S1B). Circulating melatonin levels were 60% lower in *Tph1*_*pineal*_ *−/−* mice compared to the control mice, demonstrating that Tph1‐derived serotonin is a limiting factor in pineal melatonin biosynthesis (Figure 1B). As expected, *Tph1*_*pineal*_ *−/−* or their wild‐type littermates on 129/SvJ background had undetectable levels (below sensitivity [B.S.] of the assay used) of melatonin (Figure 1B).

Histological and histomorphometric analysis of skeleton at 12 weeks of age revealed that mice lacking *Tph1* in the pineal on the melatonin‐proficient C3H/HeJ background (*Tph1*_*pineal*_ *−/−*) developed a low bone mass phenotype secondary to a decrease in osteoblast numbers and bone formation rate, while bone resorption parameters were not affected compared to +/+ mice (Figure 1C and Fig. S1C,D). μCT analysis revealed that this low bone mass phenotype in *Tph1*_*pineal*_ *−/−* mice was also present in the long bones as it was in the vertebra (Figure 1D). In contrast to the results obtained on C3H/HeJ background, mice lacking *Tph1* in pineal on melatonin‐deficient 129/SvJ background (*Tph1*_*pineal*_ *−/−* mice) had a nonsignificant change in bone mass compared to +/+ mice (Figure 1E and Fig. S1E). These results on melatonin‐deficient strain 129/SvJ (Figure 1E) underscore that Tph1‐derived serotonin produced in the pineal gland on its own has no effect on bone mass acquisition, but when it is converted to melatonin in a melatonin‐proficient strain such as C3H/HeJ (Figure 1C,D) and released in the circulation it positively regulates bone mass.

### 3.2 Exogenous melatonin administration in the WT mice increases bone mass

The above loss of function experiments revealed that PDM is a positive regulator of bone mass. We reasoned that if this is the case, then exogenous administration of melatonin in a melatonin‐deficient C57Bl/6 strain should increase bone mass. To address this question, we next fed 12‐weeks‐old female C57Bl/6 mice once daily with either vehicle or melatonin at doses 0, 10 or 100 mg/kg BW for 6 weeks (Figure 1F). Melatonin administration resulted in an elevation in the serum melatonin levels in a dose‐dependent manner (Figure 1F). Mice treated with 100 or even 10 mg/kg BW of melatonin had a higher bone mass than that of vehicle‐treated (0) mice (Figure 1F and Fig. S1F). Consistent with the influence of PDM on osteoblast proliferation and bone formation, this increase in bone mass in the melatonin‐treated mice was secondary to a major increase in bone formation parameters such as osteoblast numbers and serum osteocalcin levels (Figure 1F). In contrast to an effect on bone formation, bone resorption parameters were not affected (Figure 1F). Based on these data, it thus appears that PDM is a hormone promoting bone mass through an isolated effect on bone formation.

### 3.3 The MT2 receptor mediates melatonin regulation of osteoblast functions

If PDM acts as a hormone to promote bone mass, it must act through a specific receptor(s) present on bone cells whose inactivation should lead to a low bone mass phenotype. Among the two specific cell surface melatonin receptors, *MT1* and *MT2* both are expressed in bone (Figure 2A). MT2 receptor, however, is more restricted to the bones among the tissues analyzed, and its expression in bone was fourfold higher than that of MT1 receptors (Figure 2B). To understand which of these receptors is downstream to PDM, we performed histological and μCT analysis of the WT and mice lacking *MT1* or *MT2* on a melatonin‐proficient C3H/HeJ genetic background. Histomorphometric analysis of vertebra of *MT1*−/− mice at 12 weeks of age failed to detect any abnormalities in osteoblast number, bone formation, bone resorption and bone mass compared to +/+ mice (Figure 2C and Fig. S2A). In contrast, mice lacking *MT2* displayed a low bone mass phenotype compared to +/+ mice (Figure 2C). This low bone mass was due to a decreased osteoblast number and bone formation rate (Figure 2C). In contrast to the effect of *MT2* loss of function on osteoblasts, and like the effect of PDM melatonin, bone resorption parameters were not affected in these mutant mice compared to +/+ mice (Figure 2C and Fig. S2B). μCT analysis of long bones showed that *MT2−/−* mice had profoundly reduced bone volume, trabecular number and increased trabecular separation accounting for their low bone mass phenotype, while *MT1−/−* mice were similar to +/+ mice in all the parameters (Figure 2D). Gene expression analysis revealed a major decrease in *Cyclin* expression in *MT2−/−* mice while it remained unchanged in *MT1−/−* mice indicating a decreased osteoblast proliferation (Figure 2E). Accordingly, markers of osteoblasts differentiation were reduced in *MT2−/−* mice bone (Figure 2E). Besides, treatment of 12‐week‐old *MT2−/−* mice with melatonin at 100 mg/Kg BW/d failed to increase the bone mass (Figure 2F and Fig. S2C). These results indicate that melatonin uses one predominant receptor, MT2, to affect bone mass at both axial and appendicular skeleton.

### 3.4 Melatonin directly regulates osteoblast functions

Melatonin regulation of bone formation through MT2 receptors raises the question whether melatonin directly acts on bone cells to regulate their functions or does so indirectly. To address this issue, we used primary calvarial osteoblast cultures to identify whether melatonin regulates osteoblast functions directly.

First, we analyzed the effect of melatonin on primary osteoblasts proliferation, differentiation and mineralization. To determine if osteoblast proliferation was affected by melatonin, we measured the number of cells actively synthesizing DNA by BrdU incorporation in vitro. Melatonin significantly increased the proliferation of preosteoblasts in a dose‐dependent manner compared to the vehicle‐treated cells (Figure 3A) with a maximum increase of approximately 1.5‐fold at 100 nmol/L concentration. Analysis of osteoblasts proliferation from *MT1* and *MT2* knockout cells revealed a 50% decrease in proliferation in *MT2*‐deficient osteoblasts while *MT1*‐deficient osteoblasts proliferated similar to their WT counterparts (Figure 3B and Fig. S1A). Importantly, melatonin failed to increase proliferation in *MT2*‐deficient osteoblasts, but it raised their proliferation to the same extent as the WT cells in *MT1−/−* cells (Figure 3B). Molecularly, this increase in proliferation was partly due to the rapid increase in the expression of *Cyclins* observed in these cells upon melatonin treatment that was absent in the *MT2*−/− osteoblasts (Figure 3C,D).

We next determined whether melatonin regulates differentiation potential of osteoblasts using two different assays: alkaline phosphatase activity assays and marker gene expression analysis. Melatonin was very effective in increasing osteoblast differentiation at all the concentrations tested and ALP activity increased by 2.5‐fold at 100 nmol/L concentration (Figure 3E). Melatonin failed to increase ALP activity observed in *MT2*−/− osteoblasts (Figure 3F). At the gene expression levels, melatonin increased markers of osteoblast differentiation while it did not affect OPG/RankL ratio, an increase absent in *MT2*‐deficient osteoblasts (Figure 3G,H & data not shown).

Lastly, we looked at the effect of melatonin on the osteoblasts function by analyzing changes in mineralization in the presence and absence of melatonin. Quantitative analysis of this assay showed that melatonin increased the calvarial osteoblast mineralization in WT and *MT1−/−* by 30% (Figure 3I) at 100 nmol/L. While basal level of mineralization was decreased in *MT2−/−* calvarial osteoblasts, melatonin failed to increase it (Figure 3J,K). Further, bone marrow stromal cells from *MT2−/−* mice had a decrease (60%) in mineralization as compared to WT and *MT1−/−* mice (Figure 3, Fig. S3A,B) indicating a decrease in the number of osteoprogenitor cells in the bone marrow.

Taken together, these results demonstrate that direct action of melatonin on osteoblasts accounts, at least in part, to the overall regulation of bone mass through melatonin *MT2* pathway.

### 3.5 Melatonin treatment cures mice from ovariectomy‐induced bone loss

To assess the biological importance of the melatonin regulation of bone formation, we tested the therapeutic relevance of melatonin‐dependent increase in bone formation, through the use of ovariectomized (OVX) rodents that present, as postmenopausal women do, a decrease in bone mass.

We first tested whether melatonin treatment when given significantly late after ovariectomy can cure ovariectomy‐induced bone loss. For this purpose, 6‐week‐old mice were OVX or sham‐operated and were left untreated for 6 weeks before being treated with vehicle or melatonin at 10 or 100 mg/kg/d for another 6 weeks (Figure 4A). Osteoclast surface and serum CTX levels, a marker of bone resorption, were higher in OVX mice, regardless of their treatment, than in sham‐operated animals, and as a result, vehicle‐treated OVX mice developed a low bone mass (osteopenia; Figure 4A,B). In contrast, OVX mice treated with 10 or 100 mg/kg/d of melatonin had a higher bone mass than that of vehicle‐treated OVX mice, and at 100 mg/kg BW/d the bone mass of OVX mice was similar to that of sham‐operated mice (Figure 4A,B). Consistent with the influence of melatonin on osteoblast proliferation and bone formation, this increase in bone mass in the melatonin‐treated OVX mice was secondary to a major increase in bone formation parameters such as osteoblast numbers and bone formation rate (Figure 4B). μCT analysis of long bones showed that melatonin rescued low bone mass in the long bones as it did in the vertebrae (Figure 4C,D). We verified that serum melatonin levels and markers of osteoblasts (OCN) and osteoclasts function (Ctx) in serum were increased in melatonin‐treated OVX mice compared to sham (Figure 4E‐G). There was no change in bone length or width in any of the groups treated with melatonin (data not shown). These results establish that melatonin can rescue, through a bone anabolic mechanism, OVX‐induced osteoporosis in mice when given late after ovariectomy.

### 3.6 Melatonin treatment restores ovariectomy‐induced loss in bone quality

Ovariectomy‐induced deterioration in the bone architecture leads to a decrease in the biomechanical properties of bone or the bone quality.^33^ To evaluate the potential value of increasing melatonin levels in the treatment of low bone mass diseases, it is essential to assess whether melatonin treatment restores the deterioration in bone quality observed after ovariectomy.

To address this question, we next measured the femur stiffness and femur ultimate load, two surrogates of bone quality, upon melatonin treatment in the curative regimen. To measure bone quality, femur samples from untreated and melatonin‐treated OVX mice were subjected to a three‐point bending test and a compression analysis, to determine stiffness and maximal load (Figure 4H,I). Both parameters that were decreased after ovariectomy were restored, completely, in OVX mice treated with melatonin at 100 mg/kg/d treatment to the values seen in sham‐operated mice (Figure 4H,I). Thus, daily oral administration of melatonin could revert the bone loss, architectural deterioration and lost bone quality caused by a long‐term gonadal failure in mice through a major increase in bone formation.

## 4 DISCUSSION

**Figure 5:**
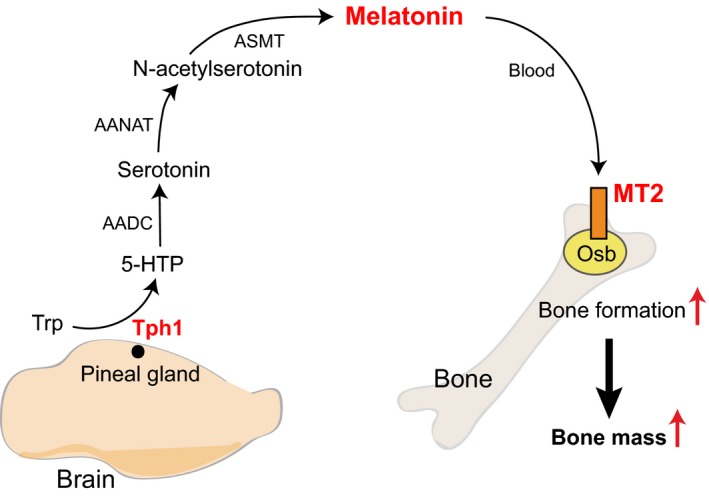
Model of melatonin regulation of bone mass. Diagrammatic representation of the proposed pathway through which pineal‐derived melatonin regulates bone mass. Tryptophan regulates through a series of reactions the synthesis of pineal‐derived melatonin (PDM) in a Tph1‐dependent manner. PDM then enters the blood stream and acts on the osteoblasts through MT2 receptors to positively regulate bone mass. Trp, tryptophan; Tph1, tryptophan hydroxylase 1; 5‐HTP, 5‐hydroxytryptophan; AADC, aromatic amino acid decarboxylase; AANAT, arylalkylamine *N*‐acetyltransferase; ASMT,* N*‐acetylserotonin *O*‐methyltransferase

This study uncovers an unanticipated molecular mechanism accounting for the melatonin regulation of bone mass. Our studies with a mouse genetic model of *Tph1* deficiency in the pineal gland using *Crx‐Cre* driver dissect the importance of pineal‐derived melatonin vs extra‐pineal synthesized melatonin in the regulation of bone mass. We further showed that one mechanism through which PDM regulates bone mass is through MT2‐receptor dependent regulation of osteoblast proliferation and function. These studies identify another tryptophan‐derived central molecule in the endocrine regulation of bone mass (Figure 5). Moreover, given the fact that melatonin and ramelteon, an MT1/MT2 receptor agonist^34^ are being used to treat sleep disorders in humans,^35^ this study provides mechanistic basis on which future therapeutic regimens could be built upon to treat bone diseases characterized by a relative or absolute decrease in bone formation.

### 4.1 Melatonin regulation of bone mass through MT2 receptor

In the present study, through pineal‐specific gene deletion and pharmacological experiments, we have demonstrated an important role of melatonin in the regulation of bone mass. In vivo pharmacological manipulations in melatonin‐deficient mice and ablation of melatonin synthesis in the pineal gland showed that melatonin profoundly affects bone mass secondary to an isolated effect on the osteoblast numbers in the bone in vivo. Investigation into the means through which melatonin affects osteoblast functions revealed that both *MT1* and *MT2* receptors are expressed on osteoblasts. According to gene deletion and in vitro experiments, MT2 is the receptor responsible for the effect of melatonin on osteoblasts, while inactivation of *MT1* does not affect bone mass. However, both in the pineal‐derived melatonin‐deficient mice and in the *MT2* null mice we did not observe any significant change in the osteoclasts parameters tested. This cellular phenotype in vivo in the bone is different than the earlier studies that have shown that melatonin affects osteoclasts function in vitro.^36^ The lack of effect in vivo could therefore point toward other indirect mechanisms at play through melatonin that offset the direct effect of melatonin on osteoclasts. However, we must point out that we have only measured few parameters of osteoclast functions and it is possible that under certain conditions and in other genetic backgrounds melatonin may affect osteoclast biology in vivo. Together, these studies reveal an important role of melatonin signaling in the regulation of bone mass through MT2 receptors in vivo.

### 4.2 Melatonin regulation of osteoblast functions

Melatonin affects multiple aspects of osteoblast biology to regulate bone mass. In vitro studies show that melatonin increases osteoblasts proliferation, differentiation and mineralization. These results are consistent with earlier studies that have shown a positive effect of melatonin signaling on the osteoblasts.^24^, ^25^, ^36^, ^37^, ^38^, ^39^ Melatonin receptors have been shown to be expressed on the osteoblasts, and previous studies have shown that melatonin positively regulates osteoblast functions.^24^, ^25^, ^36^, ^38^, ^39^ Our studies have now further extended these findings by identifying a specific receptor MT2 through which melatonin impinges on these osteoblast functions. One limitation of the present study is that we have used mice that lack *MT2* in all the cells and future studies would be needed to specifically inactivate *MT2* receptor in osteoblasts to understand the contribution of osteoblast vs other organ expression of *MT2* in the regulation of bone mass. However, the fact that *MT2*‐deficient osteoblasts show similar molecular and functional defects and are refractive to melatonin addition in vitro demonstrated that MT2 signaling in osteoblasts could play an important role in osteoblasts physiology in vivo. Another potential limitation of our results when measuring molecular basis of functional osteoblast defects is that we have only measured the steady‐state mRNA levels of the molecular markers of osteoblast functions, receptors and pineal *Tph1* expression and have not measured the protein levels. It is therefore possible that the mRNA changes in the reported genes may not translate to a decrease in the protein levels. However, our results using BrdU incorporation for the proliferation, alkaline phosphatase activity assays for differentiation, alizarin red staining that stains mineralized nodules and pineal melatonin synthesis support the conclusion that reported changes in genes expression underlie the reported defects in the cellular functions. Moreover, these results are consistent with earlier studies that have shown that Tph1 is the first step in the melatonin synthesis and that MT2 is the primary receptor responsible for the melatonin effect on the osteoblasts in vitro.^12^, ^38^

Our results, supported through systematic loss and gain of function studies with three different strains of mice (C57Bl6, C3H/HeJ and Sv129), point toward melatonin affecting bone mass through an effect on bone formation rather than resorption in the female mice in vivo. Melatonin has been shown to have different effects on the skeleton depending on the sex of the mice and the dose of melatonin utilized. In contrast to our studies on the female skeleton, it has been reported that in the male mice the mechanism used by melatonin to increase bone mass during growth is through the inhibition of bone resorption.^40^ It is important to note here that gonadal failure‐induced osteoporosis is a problem predominantly faced by females after menopause and is rare to observe gonadal failure in males. These results also underscore that evolutionarily the systems have evolved differently in males and females to respond to melatonin during growth or aging. In the ovariectomized mice, a low dose of melatonin had no effect on the ovariectomy‐induced bone loss in the rats.^41^ This is consistent with our findings wherein we did not see a statistically significant increase in bone mass in the ovariectomized mice treated with 10 mg/kg BW/d of melatonin and only saw a rescue of bone mass in the ovariectomy experiment when mice were treated with a higher dose of melatonin at 100 mg/kg BW/d (Figure 4).

### 4.3 Multiple facets of tryptophan derivatives in the regulation of bone mass

The identification of pineal‐derived melatonin as a regulator of bone mass in the present study adds another molecule to the growing list of tryptophan derivatives that regulate bone mass. Tryptophan is an essential amino acid that is converted to a variety of metabolites in different cell types to perform distinct biological functions.^12^, ^42^, ^43^, ^44^ Earlier studies have shown that in the gut, tryptophan is converted to serotonin (gut‐derived serotonin, GDS), by the rate‐limiting enzyme *Tph1*, that is a hormone and a potent inhibitor of bone formation in mice and humans.^45^, ^46^ In contrast to the GDS, brain derived serotonin (BDS) synthesized by serotonin neurons of the hind brain acts as a neurotransmitter and is a positive regulator of bone mass.^31^ Like the BDS, PDM whose precursor is tryptophan positively regulates bone mass. This preceding statement, however, needs further clarification. Although our results using *Tph1*_*pineal*_ *−/−*, MT2 receptor‐deficient and pharmacological models using melatonin‐deficient and melatonin‐proficient strains all point toward PDM playing an important role in the biology of bone, the results with the *Tph1*_*pineal*_ *−/−* mice need to be viewed in the following context. Mice that are deficient in *Tph1* in the pineal gland will be hypothetically defective in the following molecules which are known to get diffused or transported outside the cells, viz. 5‐hydroxytryptophan, serotonin, *N*‐acetyl serotonin and melatonin. All of these molecules at pharmacological doses have the potential to affect bone mass. However, melatonin treatment studies with melatonin‐deficient C57BL/6 and melatonin‐proficient *MT2* null mice on C3H/HeJ background support our conclusion that melatonin is the principle product that is involved in the regulation of bone mass in these mouse models. Together, these mouse and human genetic studies have identified an important role played by tryptophan and its metabolites in the regulation of bone mass and have illustrated that the same molecule serotonin operates through multiple organs, viz. pineal gland, gut and brain to regulate bone mass.

### 4.4 Therapeutic implications of pineal‐bone axis

The identification that PDM regulates bone mass through a specific receptor has important therapeutic implication for the following reasons. First, melatonin is a natural molecule, and its absence or abundance does not lead to any pathological state as mice that have very low/negligible or high melatonin levels are healthy and have minor differences in the physiological functions.^47^ Second, melatonin has been used successfully to cure sleep disorder at much higher doses than the one tested in the present study.^48^, ^49^ Third, the fact that pineal gland gets calcified in adult humans underscores that enhancement in melatonin levels/its receptor activity in adults might not lead to adverse consequences on physiological functions.^50^

## 5 CONCLUSIONS

Our analysis of mouse genetic and pharmacological models with and without melatonin treatment has identified a novel endocrine axis operational through the pineal gland in the regulation of bone mass. Results show that ablation of melatonin synthesis in the pineal gland or its MT2 receptors in the bone leads to a low bone mass phenotype due to an isolated defect in the bone formation. Inactivation of the *MT2* and not the *MT1* receptors leads to a cell intrinsic defect in the osteoblasts ability to proliferate, differentiate and deposit new bone matrix. Importantly, gain of function experiments revealed that oral administration of melatonin leads to an increase in bone mass in the young WT mice and is able to reverse completely the architectural deterioration and functional defects in bone in a mouse model of menopause‐induced osteoporosis. These results underscore the power of an integrative approach to bone physiology and raise several novel questions. For instance, and from a biological point of view we do not know yet whether PDM affects other endocrine axes that in turn regulate bone mass. From a therapeutic point of view, the fact that melatonin rescues ovariectomy‐induced osteoporosis in mice holds great promise given that melatonin has been used successfully to cure sleep disorders. Further experiments will be needed to determine whether pharmacologically activating MT2 using more selective receptor agonist(s) or oral melatonin administration is a valid approach in the treatment of low bone mass diseases.

## ACKNOWLEDGEMENTS

We are grateful to Dr. Gerard Karsenty for generously providing Tph1 floxed mice. This work was supported by The Wellcome Trust and the Department of Biotechnology, Government of India.

## CONFLICT OF INTEREST

Authors do not have any financial or nonfinancial competing interests with regard to the data presented in the manuscript.

## AUTHORS' CONTRIBUTIONS

VKY designed and coordinated the studies. KS performed primary cultures of osteoblasts. VKY and KS generated mutant mice. VKY and KS performed bone sectioning and the gavage studies. KS and KL stained and analyzed all the histomorphometry of the bone sections. TF provided reagents. KS and VKY wrote the paper.
