# Supplementary figures and images for: Regulation of bone mass through pineal‐derived melatonin‐MT2 receptor pathway
Source: J Pineal Res. 2017 Jun 20;63(2):e12423. doi: 10.1111/jpi.12423 (PMC5575491; doi:10.1111/jpi.12423)

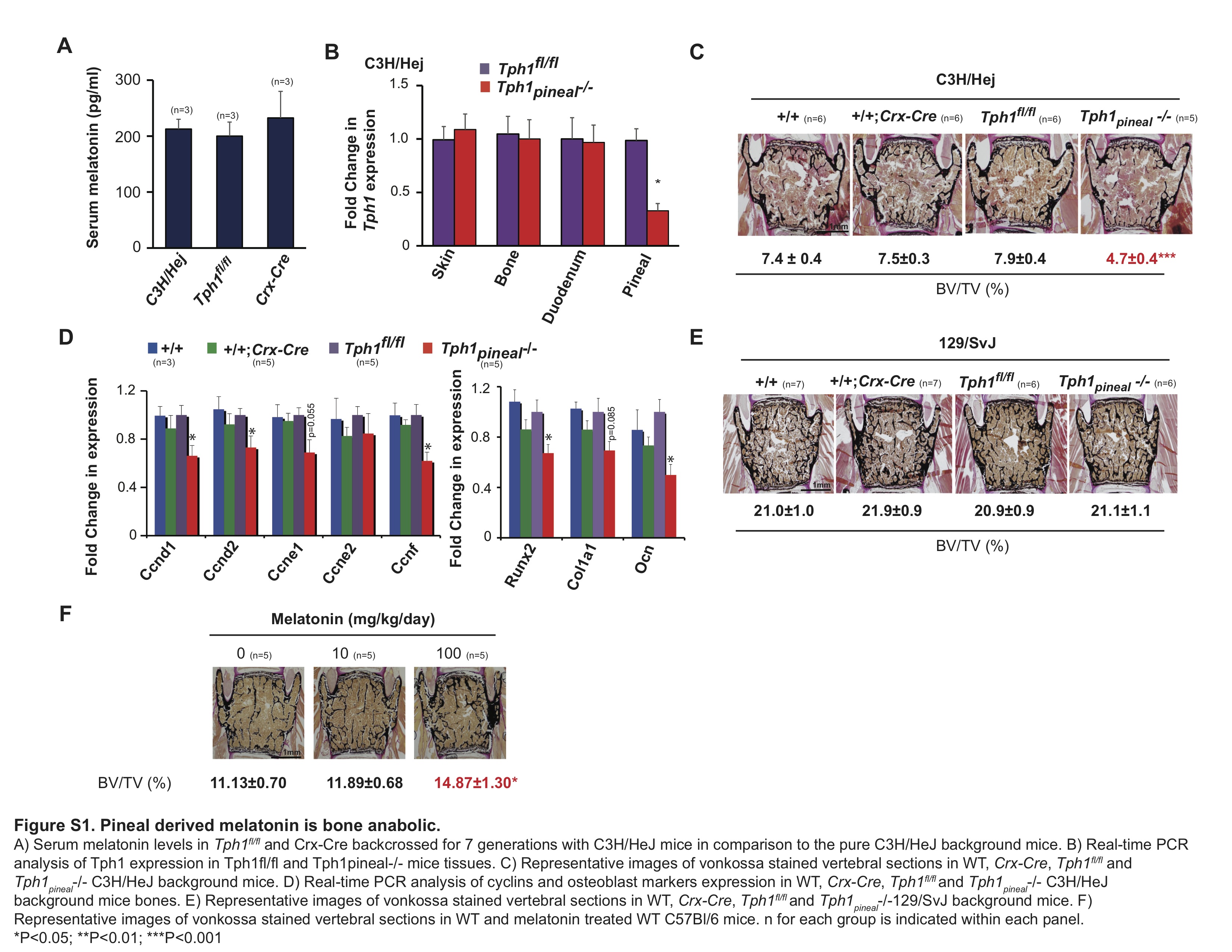

Supplement: Supplementary file 1 [file JPI-63-na-s001.jpg]

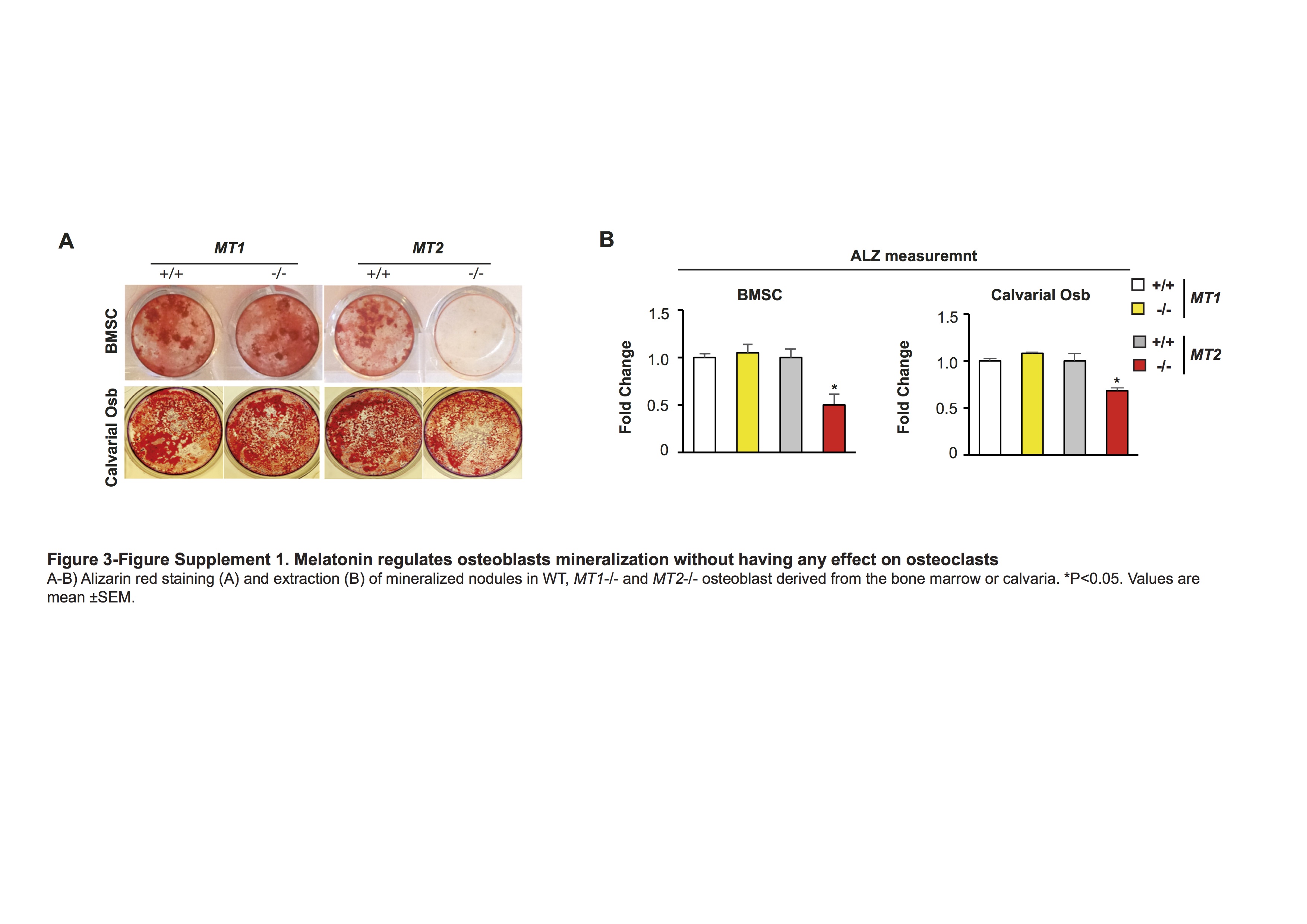

Supplement: Supplementary file 3 [file JPI-63-na-s003.jpg]
