# Supplementary material for: Regulation of bone mass through pineal‐derived melatonin‐MT2 receptor pathway
Source: J Pineal Res. 2017 Jun 20;63(2):e12423. doi: 10.1111/jpi.12423 (PMC5575491; doi:10.1111/jpi.12423)
Supplement: Supplementary file 2 [file JPI-63-na-s002.pdf]

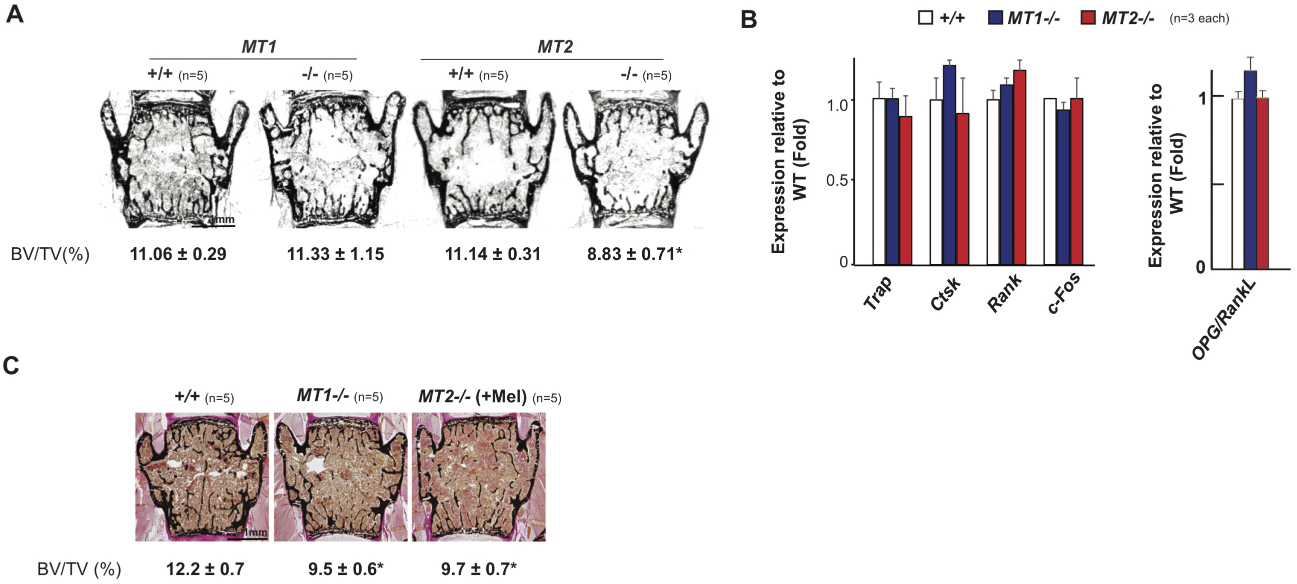

Figure S2. Melatonin acts through MT2 receptor.
A) Representative images of vonkossa stained vertebral sections of WT, *MT1*-/- and *MT2*-/- mice. B) Real-time PCR analysis of osteoclast markers expression in WT, *MT1*-/- and *MT2*-/- mice bones. C) Representative images of vonkossa stained vertebral sections of WT, *MT1*-/- and *MT2*-/- mice treated with melatonin. n for each group is indicated within each panel. *P<0.05; **P<0.01; ***P<0.001. Values are mean ±SEM.
